# An Intelligent Thermal Compensation System Using Edge Computing for Machine Tools

**DOI:** 10.3390/s24082531

**Published:** 2024-04-15

**Authors:** Endah Kristiani, Lu-Yan Wang, Jung-Chun Liu, Cheng-Kai Huang, Shih-Jie Wei, Chao-Tung Yang

**Affiliations:** 1 Department of Computer Science, Tunghai University, Taichung City 407224, Taiwan; endahkristi@thu.edu.tw (E.K.); elvawly287@gmail.com (L.-Y.W.); jcliu@thu.edu.tw (J.-C.L.); 2 Department of Informatics, Krida Wacana Christian University, Jakarta 11470, Indonesia; 3 Industrial Technology Research Institute, Hsinchu 310401, Taiwan; itria50317@itri.org.tw (C.-K.H.); itria10390@itri.org.tw (S.-J.W.); 4 Department of Mechanical Engineering, National Chin-Yi University of Technology, Taichung City 411030, Taiwan; 5 Research Center for Smart Sustainable Circular Economy, Tunghai University, Taichung City 407224, Taiwan

**Keywords:** sensor, thermal compensation, time-series model, edge computing

## Abstract

This paper focuses on the use of smart manufacturing in lathe-cutting tool machines, which can experience thermal deformation during long-term processing, leading to displacement errors in the cutting head and damage to the final product. This study uses time-series thermal compensation to develop a predictive system for thermal displacement in machine tools, which is applicable in the industry using edge computing technology. Two experiments were carried out to optimize the temperature prediction models and predict the displacement of five axes at the temperature points. First, an examination is conducted to determine possible variances in time-series data. This analysis is based on the data obtained for the changes in time, speed, torque, and temperature at various locations of the machine tool. Using the viable machine-learning models determined, the study then examines various cutting settings, temperature points, and machine speeds to forecast the future five-axis displacement. Second, to verify the precision of the models created in the initial phase, other time-series models are examined and trained in the subsequent phase, and their effectiveness is compared to the models acquired in the first phase. This work also included training seven models of WNN, LSTNet, TPA-LSTM, XGBoost, BiLSTM, CNN, and GA-LSTM. The study found that the GA-LSTM model outperforms the other three best models of the LSTM, GRU, and XGBoost models with an average precision greater than 90%. Based on the analysis of training time and model precision, the study concluded that a system using LSTM, GRU, and XGBoost should be designed and applied for thermal compensation using edge devices such as the Raspberry Pi.

## 1. Introduction

With the popularization of Industry 4.0, the manufacturing industry is gradually transforming its manufacturing mindset from mass production to customized precision manufacturing, achieving the ultimate goal of smart manufacturing. The core of smart manufacturing is the integration and application of digital and physical systems, namely cyber-physical systems (CPS) and the Internet of Things (IoT). Industry 4.0 enables high flexibility in the development, diagnosis, maintenance, and operation of automation systems. When developing these systems, people can choose the best suppliers from many components, modules, and service providers. Some diagnostics can be performed by the user, and access to “big data” helps with automation. Information can be retrieved on demand, used intelligently, and correlated to achieve automatic diagnosis. Components can be ordered automatically from the cheapest manufacturer, solving the skill shortage problem [1]. Meanwhile, recent developments show that the concept of Industry 5.0 is beginning to emerge along with the development of artificial intelligence. Data-driven decision systems help humans and robots optimize production scheduling, running equipment, forecasting breakdowns, and evaluating industrial performance. Moreover, the collaboration between humans and machines allows for a faultless environment where the versatility of humans and the precision of machines may achieve production performance that is free from errors and optimized [2].

In the post-pandemic era, the manufacturing industry uses remote access to data and cloud-based processing of large amounts of data, but this method has some drawbacks, such as data transmission delays, data storage security problems, and large-scale use of network bandwidth [3]. Therefore, the combination of intelligent manufacturing and edge computing will greatly reduce the labor cost of the manufacturing industry and improve the security of its data, while the combination of edge computing with small devices will also greatly improve the portability of the system, helping factories move toward comprehensive intelligent manufacturing [4].

In the era of global trade, product quality and product precision have become increasingly important, so it is very important to control the tooling errors of manufactured products. Among machine tool errors, thermal error is also one of the most influential factors. Excess temperature will cause thermal deformation of the machine tool. There are two main sources of machine tool thermal deformation, namely internal heat sources and external heat sources. External heat sources come mainly from solar heat radiation or the influence of ambient temperature, while internal heat sources are mainly the heat generated by the machine tool and its components during operation. The traditional method of thermal compensation is to use coolant to cool the interior of the machine, but this method does not completely improve the thermal error of the machine tool [5]. Based on reviews by Konstantinidis et al. [6], this research could be carried out at level 4 that covers advanced features such as intelligent object identification capabilities and metadata transformation techniques to address interoperability issues. In particular, classifiers with less data are typically trained via (semi)supervised machine learning. On the other hand, distributed learning techniques enhance the effectiveness of models while ensuring privacy, as seen in Federated Learning. The metadata or decisions are shown on mobile interfaces or sent to Industrial IoT platforms for further utilization or interaction with other systems.

This paper aims to use deep learning models to predict the axial displacement of machine tools and integrate them into a lightweight edge computing device that can be directly connected to the machine tool to improve the precision of the machining. The study is divided into two parts. In the first part, data recorded during machine tool operation, such as spindle temperature, speed, and torque, are used. They are used to train and predict future temperature changes. In the second part, data of multiple temperature point changes, speed changes, and corresponding five-axis displacement changes are used to train and predict the five-axis displacement. Finally, a Raspberry Pi machine tool thermal compensation system is developed to predict the future five-axis displacement. This study also develops an intelligent machine tool thermal compensation system on the Raspberry Pi Edge device. The system will collect temperature data points from the machine tool and predict future five-axis displacement. Therefore, the contributions of this study are listed as follows:

1. This study designs two experiments to develop an appropriate prediction model for time-series data on lathe machine tools.
2. This study designs a combination of genetic algorithm (GA) and the LSTM model to improve the accuracy of predicting thermal displacement in turning machine tools.
3. This study compares multiple time-series models using the same set of lathe data and integrates their final prediction results.
4. This study develops an intelligent compensation system for thermal displacement in machine tools using Qt Creator and runs the system on the edge computing side of the Raspberry Pi and the cloud computing side of Windows operating systems.

## 2. Background Review and Related Works

This section describes the relevant knowledge and literature in this study, including an introduction to edge computing, the research results of the major international companies in tool compensation, and the introduction of mathematical models of time-series models. In addition, this section provides a detailed analysis of the process and structure of genetic algorithms. More detailed information will be provided in the following section.

### 2.1. Edge Computing

With the popularity of smart manufacturing, the application of edge computing in the industry has become increasingly widespread. Ren et al. [7] introduced the design and implementation of a big data platform for intelligent industrial IoT sensor monitoring systems and predicted errors in advance. They proposed a design and implementation scheme of a manufacturing big data platform and an intelligent industrial IoT sensor monitoring system based on edge computing and artificial intelligence.

Sun et al. [8] introduced an intelligent computing architecture for IIoT with collaborative edge and cloud computing. Based on the computing architecture, they proposed an AI-enhanced offloading framework for maximizing service accuracy, which considers service accuracy as a new metric in addition to latency and intelligently distributes traffic to edge servers or remote clouds through appropriate paths. Transfer learning case studies were conducted to demonstrate the performance gain of the proposed framework.

Trinks et al. [9] introduced the most advanced edge computing in smart factories. Therefore, the results of this paper describe the priority topics of the current scientific discussion and draw the possibilities of EC to support RTA.

### 2.2. Thermal Compensation

FANUC and PFN have developed a new AI function, the AI Servo Monitor, which collects high-speed machine tool feed and spindle control data. It performs deep learning on the collected data and displays anomaly scores based on the current state of the machine components. When the machine is running normally, the AI Servo Monitor uses the motor torque data as input to train the model. The trained model extracts the features of the torque data that can represent the normal state of the torque. During actual machine operation, the AI Servo Monitor takes torque data as input, compares them with the normal state, and calculates and displays anomaly scores. This allows machine tool operators to observe symptoms of feed or spindle failure. AI Servo Monitor notifies operators to perform maintenance before a feed or spindle failure occurs, which will help improve machine availability [10].

Mazak has developed an Intelligent Thermal Shield (ITS) system [11] to address thermal displacement issues. It uses sensors placed in areas relevant to thermal displacement to collect data. For rapid and dynamic thermal displacement that occurs during axis rotation, compensation is achieved by analyzing surface displacement data based on the thermal response of the rotational speed. However, for slower and more uniform thermal displacement caused by environmental temperature changes, compensation is performed using temperature application formulas. Through similar experiments and observations, Mazak optimizes thermal displacement compensation based on the data collected by the sensors, ensuring stable and accurate machining precision under various environmental conditions. These sensors are strategically placed in locations closely related to thermal displacement.

The Advanced Process System (APS) was developed by the Swiss precision machine manufacturer Mikron [12]. The APS function is now a standard feature of Mikron’s CNC systems and can display vibration measurement signals during the machining process. APS was initially used on high-speed and heavy-duty cutting machines to monitor tool vibration and cutting force. Mikron’s HPM1100 uses a Step Tec spindle with a built-in accelerometer to measure vibration. Through this accelerometer, the current spindle vibration signal can be transmitted to the control, and with the APS function, if the received spindle vibration value exceeds a set limit, the control automatically reduces the feed rate. When the vibration value returns to normal, the feed rate can be increased again. In addition, users can view the current machine torque and vibration conditions through the control’s human-machine interface. This combination reduces vibration, increases machining accuracy, and extends the useful life of the tool. In addition to these functions, APS also includes other features, such as reducing load per unit of time, which extends spindle life, balancing holder force, and increasing machining reliability.

### 2.3. Time-Series Model

Zhou et al. [13] proposed an intelligent anomaly detection variational long-short-term memory (VLSTM) learning model based on reconstructed feature representation, which can effectively address imbalanced and high-dimensional problems in industrial big data and significantly improve the accuracy of data anomaly monitoring while reducing the error rate in the industry. Ren et al. [14] proposed a data-driven self-supervised long- and short-term memory deep factorization machine (LSTM-DeepFM) model driven by data for soft industrial measurement, including a framework of pre-training and fine-tuning stages to explore different features of industrial data. Mateus et al. [15] used an LSTM model to predict the future condition of industrial paper machine equipment based on sensor data, maximizing industrial plant maintenance and supporting decision-making about equipment availability. Alazab et al. [16] proposed a novel multidirectional long-short-term memory (MLSTM) technique to predict the stability of intelligent power grid networks and compared this model with other related time-series models. The results showed that the MLSTM method outperformed other ML methods.

Liu et al. [17] proposed a multifactor installed capacity prediction model based on bidirectional long- and short-term memory-gray relational analysis to predict the installed capacity of solar photovoltaics. The results showed that the prediction accuracy of the GRA-BiLSTM model was higher than that of other models. Lan et al. [18] proposed a method called threshold optimization, which combined the CNN-BiLSTM-Attention model with a threshold modification method based on receiver operating characteristic (ROC) curves. The experimental results showed that this method can improve the accuracy (AC) and the minority class detection rate (DR) at low false alarm rates (FR), outperforming other intrusion detection methods. Prihatno et al. [19] developed a single dense layer bidirectional long-short-term memory (BiLSTM) model to predict PM2.5 concentrations in indoor environments using time-series data. The method achieved high precision with low errors in predicting PM2.5 concentrations in the cleanroom of a semiconductor factory.

Cavdar et al. [20] proposed a method that combined 1D convolutional neural networks (1DCNN) and the Dempster-Shafer (DS) decision fusion approach (DS-1DCNN) for anomaly decision-making in IIoT. Based on the simulation results obtained, this method improved the accuracy of the decision and significantly reduced uncertainty. Compared to Long-Short-Term Memory (LSTM), Random Forest, and CNN models, the proposed method demonstrated superior performance. The average recall rate was 0.9763, and the average precision was 0.9899 on the Mill dataset, indicating acceptable and reliable decision outcomes.

### 2.4. Related Works

Liang et al. [21] present a new system to predict thermal error on heavy-duty CNC machines. The methods used were LSTM networks and fog–cloud architecture. The results indicate that, compared to procedures without employing the intended system, the system reduced the amount of data transferred by 52.63% and increased the precision of the machining by 46.53%.

Gui et al. [22] considered the prediction and control of the error of the spindle system thermal problems; a new mist–edge fog–cloud system (MEFCS) design is recommended. The methods were Bi-LSTM network and cosine and sine gray wolf optimization (CSGWO) algorithms. The results show that the accuracy level of the deviation of the tooth profile is increased from ISO level 5 to ISO level 3 with the suggested MEFCS. The mist-cloud structure, mist-edge-cloud structure, mist-cloud structure, and mist-edge-cloud structure, respectively, have execution times of 206 s, 200 s, 186 s, and 167 s.

Guo et al. [23] study the spatiotemporal correlation of data in the static thermal deformation modeling of CNC machine tools. The methods use a hybrid CNN-LSTM model with spatiotemporal correlation (ST-CLSTM). The results of the ST-CLSTM model are great, robust, and have good prediction performance. The ST-CLSTM model outperforms other comparison models in terms of prediction accuracy, generalization ability, and robustness in three different directions through thermal error studies conducted under various situations (such as varying spindle speed and ambient temperature).

Kuo et al. [24] discuss an approach for autonomous optimization using bidirectional GRU that can accurately forecast manufacturing mistakes. The methods implement an optimized automatic logistic random generator time-varying acceleration coefficient particle swarm optimization (LRGTVAC-PSO) method to optimize a branch structured bidirectional Gated Recurrent Unit (GRU) neural network. The results for issues requiring prediction related to time bidirectional GRU produce better results. The accuracy of the suggested method is higher than that of the other optimized algorithms analyzed in this study, with a three-axis average of 0.945.

Kuo et al. [25] use sophisticated algorithms to forecast the thermal displacement of the machine tool. The methods apply an ensemble model to integrate long short-term memory (LSTM) with a support vector machine (SVM). The experimental findings demonstrate that LSTM-SVM has better prediction performance than other machine-learning methods. The experiment shows that the prediction error RMSEs were successfully reduced to 2.13, 3.91, and 2.04 using this hybrid LSTM-SVM model. The overall mean RMSEs are 2.69, which is better than 3.28 and 2.97, respectively, for the LSTM and SVM models.

Liu et al. [26] determine the relationship between spindle thermal errors and temperature fluctuations. This study aimed to build a reliable and efficient spindle thermal displacement modeling method. The algorithms applied the comparison of three types of modeling methods: LSTM, MLR, and BPNN. The results show that, especially under high rotation spindle speed, the performance of the ANN-based modeling schemes (LSTM and BPNN) significantly surpassed the MLR modeling method. At all spindle operating circumstances, the suggested LSTM has a lower root mean square error (RMSE) than a BPNN. The suggested spindle thermal error prediction technique is validated at spindle rotation speeds of 3000, 6000, and 9000 rpm.

Liu et al. [27] performed spindle systems, thermal error modeling, and compensation based on the error mechanism of spindle systems. The methods used were the VMD-GW-LSTM network, the VMD-LSTM network, and RNN. According to the findings, the compensation rates for sizes 1, 2, and 3 of the VMD-GW-LSTM network model are 77.78%, 75.00%, and 77.78%, respectively. Furthermore, compared to the VMD-LSTM network and RNN models, the VMD-GW-LSTM network model performs both prediction analysis and compensation analysis far better.

Nguyen et al. [28] reduce the thermal error of the workpiece. The study builds a thermal deformation prediction model using an artificial neural network and applies real-time error correction to a three-axis vertical CNC milling machine in cutting processes. The methods apply LSTM with Pearson’s correlation coefficients for feature selection. This study demonstrates how well a real-time error correction system for a CNC milling machine may function when an LSTM neural network is used as the temperature error prediction model. With real-time error compensation, the thermal error on the X-axis reduced from 7 to 3 m, the thermal error on the Y-axis decreased from 74 to 21 m, and the thermal error on the Z-axis decreased from 64 to 20 m during an 8-h cutting experiment, according to the dimensions of the workpiece.

Zeng et al. [29] predicted thermal error and were controlled by an edge cloud system. The methods implement a Sequence-to-Sequence model-based LSTM network with an attention mechanism (SQ-LSTMA). The results show that the SQ-LSTMA model outperforms other networks in terms of prediction performance and convergence rate. Additionally, the calculation time is shortened as a result of the attention mechanism. The respective prediction accuracy of the BP, RNN, LSTM, SQ-LSTM, and SQ-LSTMA models is 90.98%, 97.66%, 95.32%, 95.37% and 99.02%. The computation times for the BP, RNN, LSTM, SQ-LSTM, and SQ-LSTMA models are, respectively, 10.36 s, 17.94 s, 100.50 s, 80.81 s, and 41.05 s.

Ji et al. [30] introduce an innovative deep neural network topology that efficiently utilizes multisensor information to achieve reliable perception in unorganized and unpredictable environments. This method uses the intended trajectory and the present sensor observation to estimate the likelihood of future failure. The detector achieved superior performance in detecting navigation failures in agricultural environments using a feature-level camera-lidar fusion. It outperformed other state-of-the-art approaches in terms of F1-score and PR-AUC. In the real-time test, they showcased the proposed proactive anomaly detection network (PAAD), which has a reliable ability to detect anomalies with minimal false alarms.

Lee et al. [31] propose a lightweight and efficient solution named Realtime Ready to go (ReRe) that can accurately identify anomalies in real time. The suggested system adaptively modifies its two long-term detection thresholds over time and retrains its two LSTM models as needed. The results indicate that the suggested system performs comparably well with real-time time-series anomaly detection (RePAD), AnomalyDetectionTs (ADT), and AnomalyDetectionVec (ADV).

Gupta et al. [32] introduced supervised prediction classification methods. In the first stage, they produce forecasts for future resource levels. In the second step, they analyze these predictions to identify any anomalies. The proposed approach combines LSTM and BLSTM models to forecast future resource usage trends and detect anomalies in cloud workloads in advance. The performance of various resource prediction models, including RNN, LSTM, bidirectional LSTM, and a hybrid of LSTM and BLSTM, was tested. The hybrid model was shown to have the highest performance.

Spantideas et al. [33] present a practical application of a system consisting of three components (MCS server, NWDAF, and Orchestrator) within the context of MCS services. The purpose of this system is to identify the occurrences of MCS overload and to facilitate the efficient allocation of computational service resources. Additionally, it is important to predict future requirements and notify the orchestrator to take proactive measures in relation to the scalability of the service.

Wang et al. [34] introduced an innovative method for predictive and proactive maintenance in the field maintenance of HSR power equipment. The LSTM-RNN-driven maintenance predictor has demonstrated its remarkable capability to forecast future maintenance times using historical sample data.

Psarommatis et al. [35] proposed faultless manufacturing by optimizing efficiency and effectiveness in production processes. The study used machine vision technology, demonstrating its superiority over traditional methods in real-world situations. The showcase used simulation to show the enhancement of in-line process systems’ performance with the implementation of machine vision. Furthermore, the article addressed significant obstacles in the implementation process, including the management of environmental contamination, the optimization of machine coordination, the accommodation of various part sizes, and the configuration of efficient coolant delivery systems. The comprehensive examination of crucial elements includes the durability of machine vision equipment, training for operators in machine vision technology, and a cost-benefit analysis of its adoption. The research highlights the crucial role of machine vision in revolutionizing production settings and improving advanced automation systems.

## 3. Research Methodology and Framework

In this section, the research framework of the study will be introduced, which includes a detailed description of the GA-LSTM model used in Experiment 2.

### 3.1. Research Framework

Research processes have two main steps. First, feasible time-series variations are investigated based on the data collected of time, speed, torque, and temperature changes at different positions of the machine tool. Subsequently, using the identified feasible machine-learning models, the study analyzes different cutting conditions, temperature points, and speeds of the machine to predict the future 5-axis displacement. To ensure the accuracy of the models developed in the first step, additional time-series models are explored and trained in the second step, and their performance is compared with the models obtained in the first step.

**Figure 1:**
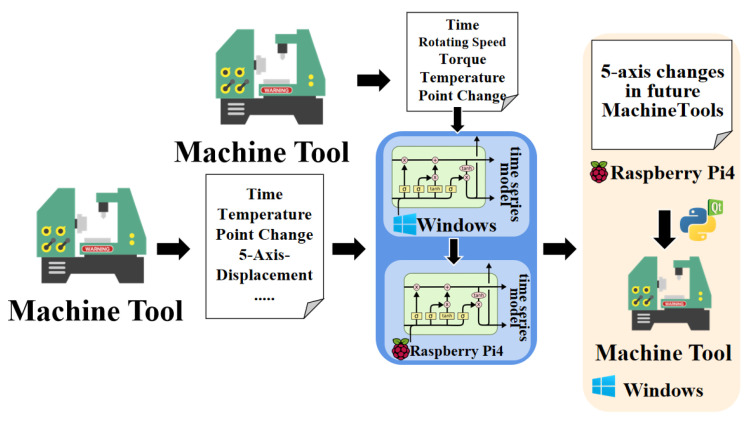
Complete Research Framework.

Upon completion of the aforementioned research, a human-machine interface is built using Qt Creator in both Raspberry Pi and Windows environments, allowing users to predict real-time thermal displacements of the machine. Figure 1 illustrates the complete research framework.

### 3.2. Data Preprocessing

Due to the large number of temperature points in the data set, the prediction of the model may suffer from overfitting. Therefore, in this experiment, Pearson’s correlation analysis was performed to select the eight top temperature points that have a significant contribution to the displacement of the five axes. Pearson’s correlation analysis is used to explore the linear relationship between two continuous variables (*x* and *y*). If the absolute value of the correlation coefficient between the two variables is large, it indicates a high degree of mutual covariance. Generally, if the two variables are positively correlated, an increase in *x* will correspond to an increase in *y*. On the contrary, if the two variables are negatively correlated, an increase in *x* will result in a decrease in *y*. The formula for the Pearson correlation coefficient is as follows, where *r* represents the correlation coefficient and COV is the covariance.

(1) $$r\left(x,y\right)=\frac{COV(x,y)}{SxSy}=\frac{\sum_{i=1}^{n}(xi-\bar{x})(yi-\bar{y})}{\sqrt{\sum_{i=1}^{n}((xi-\bar{x}{)}^{2})\sum_{i=1}^{n}(yi-\bar{y}{)}^{2}}}$$

### 3.3. AI Model for Experiment 1

This experiment used the LSTM, GRU, TCN, and Ensemble Stacked models. Let us provide an overview and description of each model’s architecture.

The LSTM model requires the input data to be in a specific format. In this study, the data were reorganized to meet the input requirements of the LSTM model. The data were transformed into groups of 10 records, with each record consisting of 5 attribute values. The input data were then reshaped into the format [*n*, 10, 5], where *n* represents the total number of samples. The output data were shaped as [*n*, ].

The GRU model requires that the input data be in a specific format. In this study, the data were reorganized accordingly. The data were transformed into groups of ten samples, each sample consisting of five attributes. The input data were reshaped in the shape of [*n*, 10, 5], where *n* represents the total number of samples. The output data had the shape of [*n*, ].

The TCN (Temporal Convolutional Network) model also requires that the input data be in a specific format. For this study, the data were reorganized accordingly. The data were transformed into groups of ten samples, each sample consisting of five attributes. The input data were reshaped in the shape of [*n*, 10, 5], where *n* represents the total number of samples. The output data had the shape of [*n*, ].

### 3.4. AI Model for Experiment 2

In addition to the time-series models used in Experiment 1, this experiment also included the training of seven additional time-series models of different types. They are WNN, LSTNet, TPA-LSTM, XGBoost, BiLSTM, CNN, and GA-LSTM. The following describes its model content.

The usage condition for the BiLSTM model is that the input data should adhere to the input format of the BiLSTM model, restructuring the data set accordingly. For this experiment, the data were transformed into groups, each group consisting of one record that contains nine attribute values. The input data were then reshaped into the format [*n*, 1, 9], where n represents the total number of records. The output data format is [*n*, ].

The usage condition for the LSTNet model is that the input data should conform to the input format required by the LSTNet model. It necessitates reorganizing the dataset accordingly. For this experiment, the data were transformed into groups, each group consisting of 20 records that contain nine attribute values. The input data were then reshaped into the format [*n*, 20, 9], where n represents the total number of records. The output data format is [*n*, ].

The usage condition for the 1DCNN model is that the input data should conform to the input format required by the 1DCNN model. Reorganizing the data set accordingly is necessary. For this experiment, the data were transformed into groups, with each group consisting of one record containing nine attribute values. The input data were then reshaped in the format [*n*, 1, 9], where n represents the total number of records. The output data format is [*n*, ].

After multiple experiments, it was determined that for this experiment, XGBoost should be trained and used for predictions using the multivariate regression approach. Sklearn provides a wrapper class called MultiOutputRegressor that meets the requirements of this experiment. However, it is necessary to configure the parameters of MultiOutputRegressor accordingly.

### 3.5. GA-LSTM

In the second experiment, this study proposes a recurrent neural network GA-LSTM (Genetic Algorithm-Long-Short-Term Memory) to predict the displacement of the five axes based on the previous experimental results. Transforms the problem into genes using binary representation, combines multiple sets of genes into a population, and calculates the fitness of each chromosome in the population using the fitness function. Based on fitness, better chromosomes are selected for crossover and mutation to obtain solutions that are closer to the optimal solution. Iteration continues until the termination condition is satisfied.

This study combines GA (Genetic Algorithm) with LSTM (Long-Short-Term Memory) and uses GA to optimize the parameters of the LSTM training process. The main parameters optimized by GA include the data time steps (look_back, lb), the hidden layers of the LSTM model (lstm_nets, ls), the number of training epochs (epochs, ep), and the dropout rate (dp). After obtaining the optimal parameter set, the five-axis displacement dataset of the machine tool is used as input data, and the predicted values of the machine tool’s five-axis displacement are used as the output matrix. The model weights are adaptively adjusted, and the GA-LSTM model is constructed. The data set is trained using this model, and the predicted values are compared with the actual values.

## 4. Experimental Procedures

### 4.1. Experimental Environments

**Figure 2:**
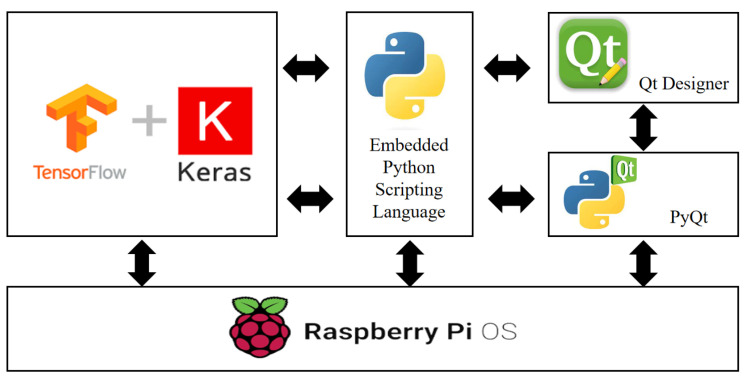
System Environment Architecture Diagram.

This study mainly involved training and prediction using a general computer and a Raspberry Pi. The hardware of the general computer utilized the VACS (Virtual AI Computing System), which includes the Nvidia Quadro RTX8000 GPU (NVIDIA, Santa Clara, CA, USA) for testing purposes. For the Raspberry Pi (Raspberry Pi Foundation, Cambridge, UK), two units of the 4B 8 GB RAM version were used. The operating system used was Raspberry Pi OS (64-bit), Debian version: 12 the official operating system provided by Raspberry Pi. This system is based on Debian Linux. One of the Raspberry Pi units served as the system monitoring host, while the other unit was installed with AI packages for the main testing and computation tasks. The system of this study is primarily implemented on Raspberry Pi 4 with 4 GB RAM hardware. The user interface is developed using QT Creator 4.14. Python is utilized on this platform to build AI models. Various functionalities are executed by calling Python functions through QT Creator. Figure 2 represents the architecture diagram of the system environment.

### 4.2. Experimental 1

#### 4.2.1. Dataset Introduction

Based on our research on smart machine tools from international controller manufacturers such as Fanuc and Siemens and machine tool manufacturers such as DMG, Okuma, Mazak, and Mikron, we have found that there is limited publication and literature available on prediction studies specifically focused on rotary axes. Most of the major manufacturers mainly focus on thermal compensation for machine tools in their research and discussions. Additionally, we have observed that many manufacturers have developed new smart machine tools, but our search for the core technical literature on these types of machine tools did not reveal any related predictions regarding rotary axes. Therefore, in this study, we have selected models that demonstrate better training performance in the field of time-series prediction based on the collected time-series dataset.

**Figure 3:**
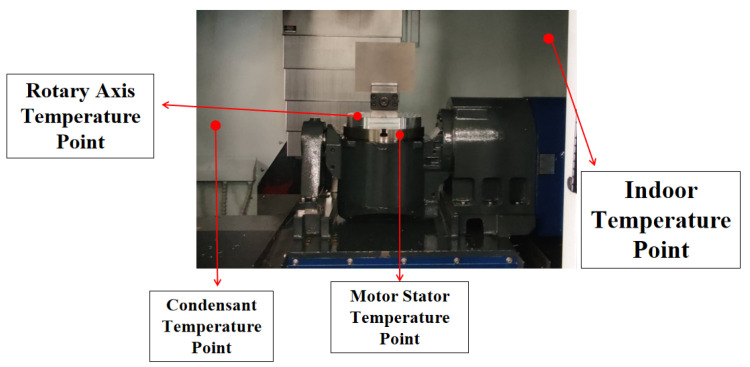
Temperature Point Display.

The acquisition of the data set for this study mainly involved the collection of data on-site from the operating machine tools. We capture real-time speed and torque data from machine tools and save it. We used an infrared temperature sensor to measure and record temperatures at key points of the machine tool, as shown in Figure 3. The data set for this study mainly collected four temperature points, namely indoor temperature, condenser temperature, rotary axis temperature, and motor stator temperature. We recorded the variations of these four temperature points every minute during the experiment.

**Figure 4:**
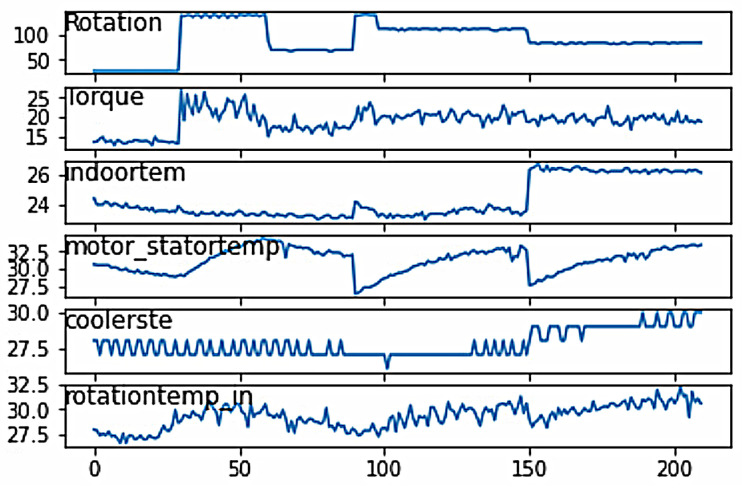
Temperature Line Chart.

To ensure that the prediction results align closely with the actual working conditions of the machine tool, we observed temperature variations by introducing changes in speed. From the line graph, it is evident that there are slight changes in the temperature trend with variations in speed, as shown in Figure 4.

#### 4.2.2. Data Preprocessing

In this study, we selected a time difference of one minute for each data point. After normalizing the data, we transformed the time-series problem into a supervised learning problem using a sliding-window approach. According to the research proposal, the task requires predicting the rotary axis temperature for the next ten minutes based on the previous ten minutes of data. The input values consist of the motor stator temperature, speed, torque, indoor temperature, and condenser temperature at time t − 1, while the output value is the temperature on the inner side of the rotary axis at time t + 9.

Regarding data preprocessing, there are two main aspects of data cleansing. The first part involves checking the consistency of the data, specifically handling outliers or values that are too large or too small, to ensure that the data align with realistic values. The second part involves handling invalid or missing values in the data. In our study, data were collected by measuring on-site, and we performed simultaneous checks and processing for both aspects during the data collection process. Therefore, there is no need for further data cleansing in the data set used in this investigation.

**Figure 5:**
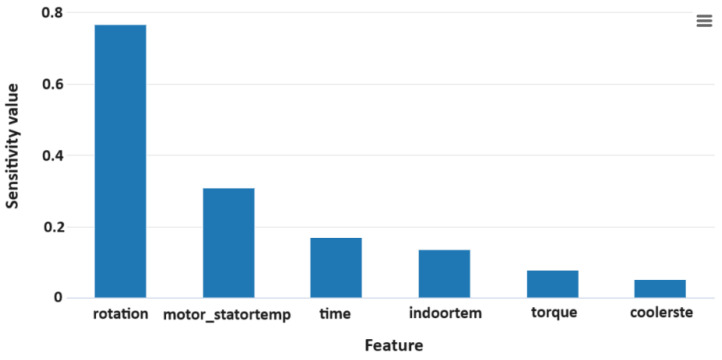
Attribute Sensitivity Analysis Chart.

During the data collection process, we collected six attribute values that can affect the temperature variation on the inner side of the rotary axis. To understand the correlation between attributes and temperature variation, we used Microsoft Azure to perform sensitivity analysis on the dataset used in this investigation. Microsoft Azure [36] is a Microsoft-provided public cloud service platform that offers AutoML capabilities for a complete automated analysis of datasets. The results of the sensitivity analysis for this research are shown in Figure 5. It can be observed that the attributes with the greatest impact on the temperature variation on the inner side of the rotary axis are the speed and the motor stator temperature.

### 4.3. Experimental 2

#### Dataset Introduction

**Figure 6:**
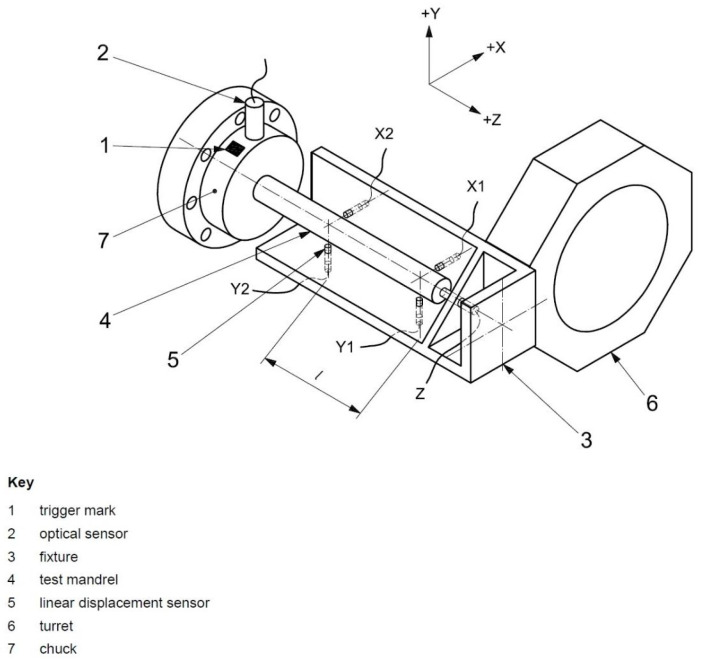
Five-axis Machine Tool or Five-axis Machining Center Display.

This dataset was provided by the Industrial Technology Research Institute and focuses mainly on the temperature variations and five-axis displacements of two different machine tools under different conditions. Five-axis machining is a machining mode of CNC machine tools that utilizes the linear interpolation motion of the X, Y, Z, A, B, and C axes. Machine tools used for five-axis machining are commonly referred to as five-axis machine tools or five-axis machining centers, as shown in the following Figure 6. The machine possesses the capacity to execute tool or workpiece displacement along five distinct axes concurrently. These axes are denoted by different arrows labeled X, Y, Z (for linear displacement) and A, B (for rotational displacement). The + and − symbols indicate the direction of movement along each axis. This capability enables the execution of complicated machining operations, such as drilling, milling, and tapping, at various angles without necessitating the repositioning of the workpiece.

As shown in Table 1, the first machine tool provides datasets for three different conditions, comprising a total of 41 columns. These columns include time, rotation speed, 34 temperature points, and displacement variations of the five axes. On the other hand, the second machine tool provides datasets for four different conditions, comprising a total of 60 columns. These columns include time, rotation speed, 53 temperature points, and displacement variations of the five axes.

**Figure 7:**
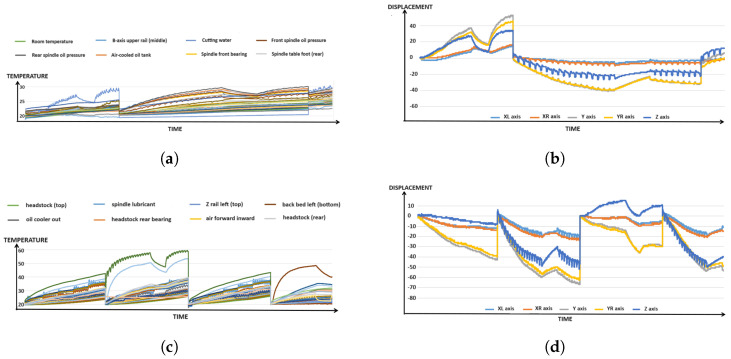
The different operating conditions of the machine tool. (**a**) Tool 1 Temperature Line Chart. (**b**) 5-axis Displacement of Tool 1. (**c**) Tool 2 Temperature Line Chart. (**d**) 5-axis Displacement of Tool 2.

The experiment ultimately integrates the different operating conditions of the machine tool into a single dataset to increase the amount of data available for model training. Figure 7a displays the temperature variations at different points for Tool 1 in a line chart, while Figure 7b shows the corresponding displacement of the five axes. Similarly, Figure 7c represents the temperature variations at different points for Tool 2, and Figure 7d illustrates the corresponding displacement of the five axes for Tool 2.

## 5. Research Results and Discussion

### 5.1. Experiment 1 Results

**Figure 8:**
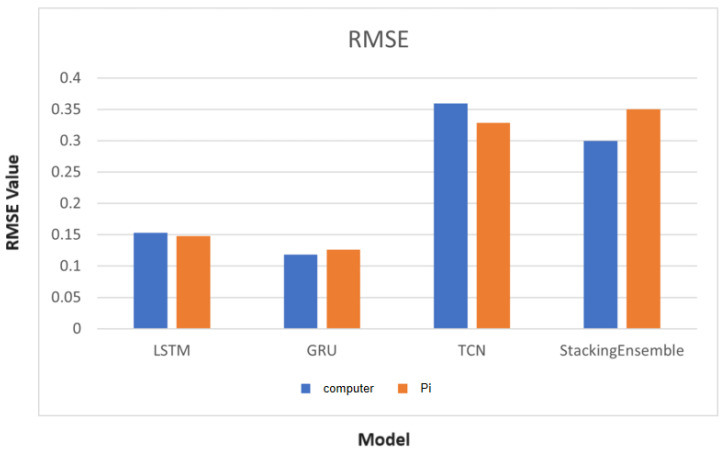
Histogram of RMSE Results of AI model.

The dataset for Experiment 1 consists of a total of 300 records. In this study, the data set is divided into a training set and a test set in an 8:2 ratio for model training. To evaluate the prediction results of the aforementioned models, we mainly use RMSE (Root Mean Square Error) to check if they meet our requirements, as shown in Figure 8. It can be observed that both the GRU and LSTM models yield lower RMSE values, indicating that these two AI models provide more accurate predictions compared to actual values based on the collected data set.

Although the TCN model also achieves an RMSE below 1 in most experiments, its performance is comparatively poorer than that of the LSTM and GRU models. In some cases, the TCN model does not converge during training, leading to increased instability in the predictions.

In addition, although the Stacking Ensemble Learning algorithm performs well in overall prediction, we have observed that its results can vary significantly when different rotational speeds are provided for prediction. Therefore, if the machine tool operates with a fixed set of rotational speeds, this AI model might be a good option due to its faster training speed and higher accuracy. However, if the machine tool requires a wide range of rotational speed changes, it is not recommended to use this model due to its inconsistency in predictions.

**Figure 9:**
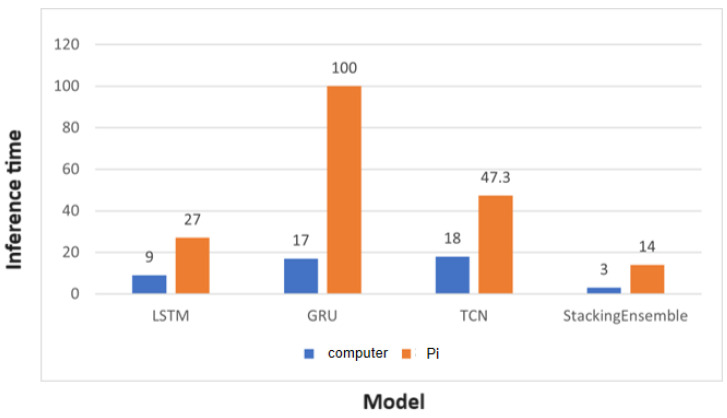
Histogram of Computing Time between General Computer and Raspberry Pi.

In addition, we compared the runtime of these four models on the Raspberry Pi, as shown in the above Figure 9. It can be observed that the inference time on the Raspberry Pi is significantly longer compared to a general computer (including the GPU). However, considering that the Raspberry Pi is a lightweight and relatively inexpensive product compared to a general computer, it can be considered a lightweight edge AI computing tool, especially when computational time is not a critical requirement.

### 5.2. Experiment 2 Results

**Figure 10:**
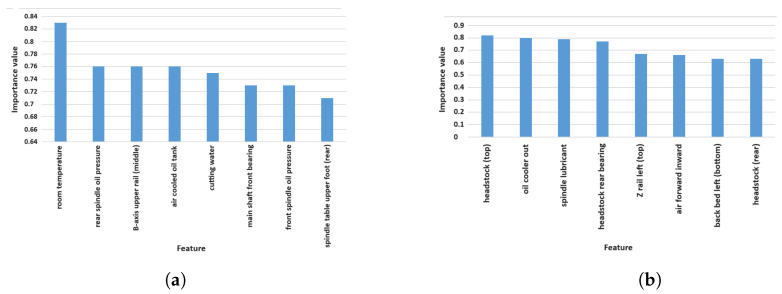
Histogram of the eight temperature points. (**a**) Tool 1. (**b**) Tool 2.

For Experiment 2, Machine Tool 1 has a total of 6433 datasets, and Machine Tool 2 has a total of 7423 datasets. In this study, each data set is divided into a training set and a test set in an 8:2 ratio for model training. According to Pearson’s correlation analysis, Figure 10a shows the top eight temperature points and their corresponding importance values in relation to the five-axis displacement for Tool 1, and Figure 10b shows the top eight temperature points and their corresponding importance values in relation to the five-axis displacement for Tool 2. In the inspection by experts in the field, these results align well with their practical experience.

Based on the sensitivity analysis, we filtered the data set for the first eight temperature points related to the machine tool. We then trained the selected dataset using the prebuilt deep learning models. To evaluate the prediction results in this study, we adopted the determination coefficient ($R^{2}$ score) as an assessment metric. In regression models, this coefficient mainly reflects the accuracy of model predictions compared to actual values. A higher coefficient indicates a higher accuracy of the model predictions, with values ranging from 0 to 1. The calculation formula for $R^{2}$_score is as follows:

(2) $$R^{2}\left(y,\hat{y}\right)=1-\frac{\sum_{i=0}^{nsample-1}{\left(yi-\hat{y}i\right)}^{2}}{\sum_{i=0}^{nsample-1}{\left(\hat{y}i-yi\right)}^{2}}$$

**Figure 11:**
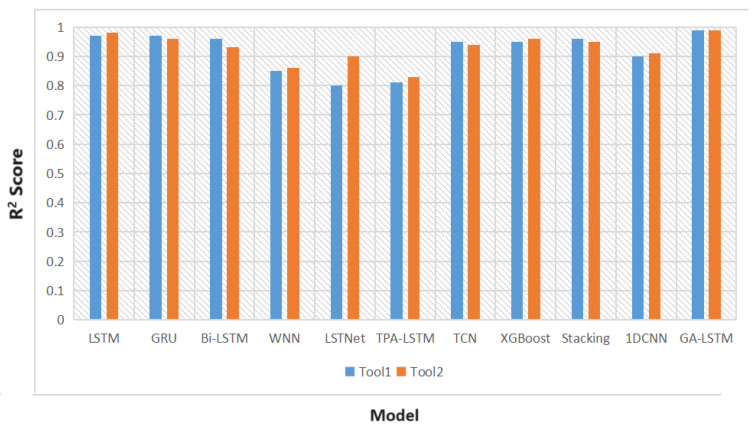
Histogram of Experimental Research Results.

As shown in Figure 11, it can be seen that, under the same dataset and hardware environment, the research results of this experiment indicate that the time-series models used in this study have achieved a $R^{2}$ score of 0.8 or higher. The GA-optimized LSTM model developed in this investigation also demonstrates excellent performance with an accuracy of 0.99.

**Figure 12:**
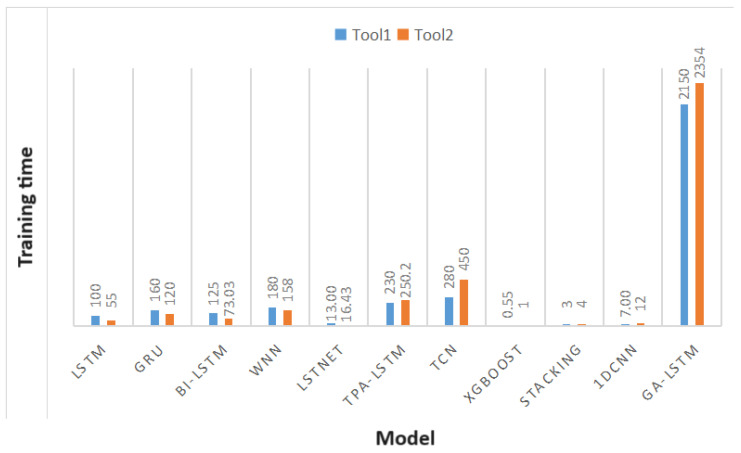
Histogram of Model Training Time.

In the early stages of system development, we evaluated the overall performance of AI models. Figure 12 shows the training times for each model on a general computer. It can be seen that although GA-LSTM achieved the highest prediction accuracy, it required a relatively longer training time. Compared to GA-LSTM, the LSTM, GRU, and XGBoost models, it provided overall more suitable results for practical applications.

### 5.3. Discussion

**Figure 13:**
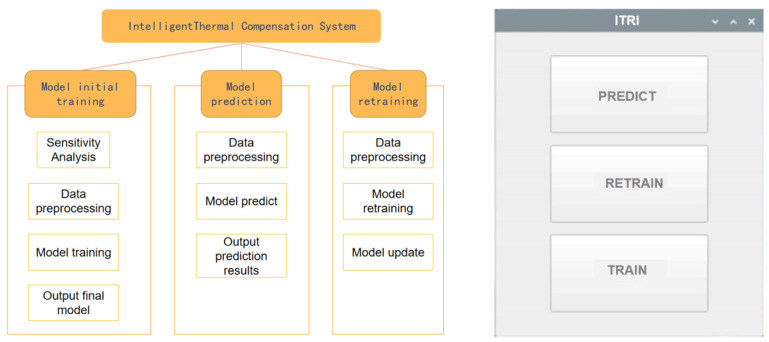
Schematic Diagram of Platform Functions.

Based on the entire experiment, the development of the system in this study consists mainly of three main functionalities: model training, model prediction, and model retraining, as shown in Figure 13.

The first functionality of the system is the initial training of the model. The system performs sensitivity analysis on the user input data set to identify the top 8 temperature points that have the greatest impact on the 5-axis displacement. The data are then preprocessed, specifically for the 8 selected temperature points. The system calculates the temperature differences by subtracting the initial temperature values from the selected temperature values. The dataset is further split to suit the input format of the AI models.

**Figure 14:**
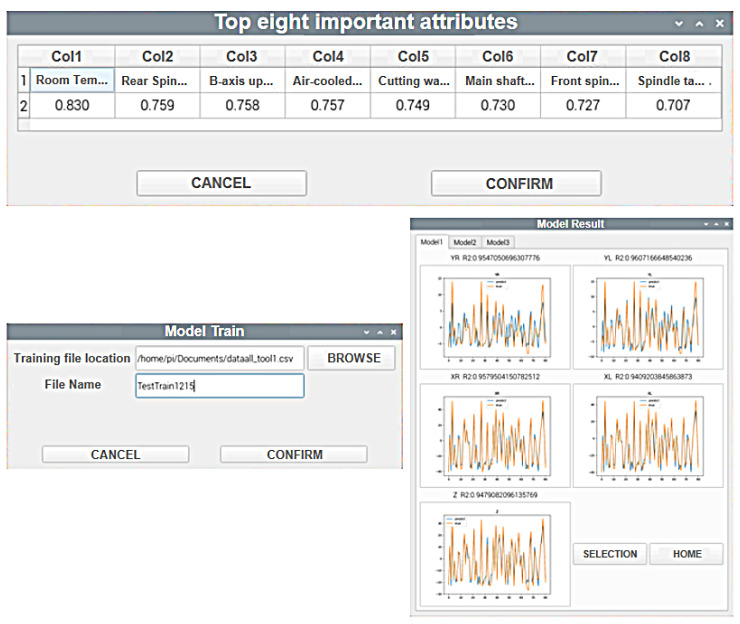
Raspberry Pi Interface 1.

Next, the system trains and validates the input data set using three different AI models. It outputs the predictions of the three models, along with the corresponding $R^{2}$_score and a line chart that compares the predicted values and the actual values. These results are provided to the user for selection. Based on the experience, the user can choose the most suitable model for the specific machine tool. Finally, the selected model is exported and made available to the user. The Raspberry Pi interface of the system is shown in Figure 14.

**Figure 15:**
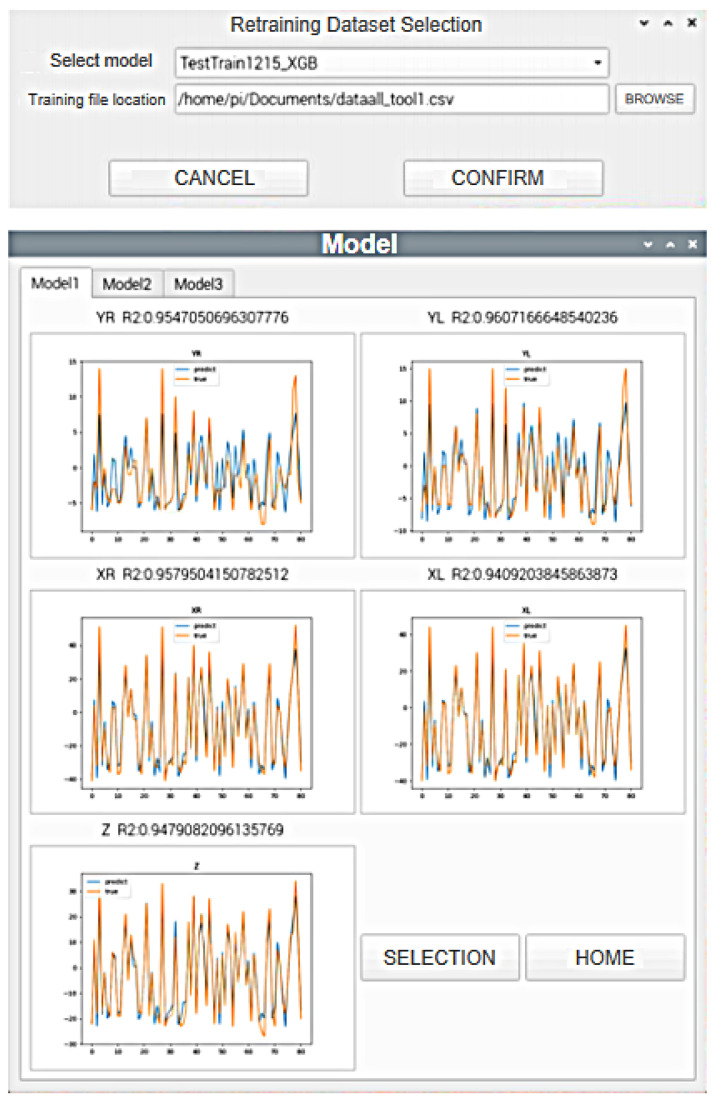
Raspberry Pi Interface 2.

The second functionality addresses the need to retrain the models as the dataset evolves over time. The platform provides the ability to retrain the models by preprocessing the new dataset and using the models obtained from the first part. The updated models are then outputted for further use. The Raspberry Pi interface for this functionality is shown in Figure 15.

## 6. Conclusions

Based on the two experiments conducted, it was observed that applying different time-series models to the machine tool dataset yielded favorable prediction results. Furthermore, the GA-optimized LSTM model developed in the second experiment achieved the best performance. In future research, efforts will be made to optimize the training time of the GA-LSTM model, and additional algorithms will be applied to analyze multiple temperature points in the machine tool compensation data set. The goal is to identify the recommended features and integrate them into the developed Raspberry Pi smart compensation system. Based on the research results, three AI models were selected and developed for edge computing terminals, which demonstrated good performance. Additionally, the operating system was modified to enable its use in the machine cloud at the Industrial Technology Research Institute. Finally, the precision of the research models reached more than 0.96, which is in line with the current trend of using deep learning in the manufacturing industry to improve the thermal errors of the machining. Although this research has achieved a high accuracy of over 0.96 in predicting the five-axis displacement of machine tools, further improvements are needed to apply the developed system to real factory machines. For example, in this study, data were provided by selecting file paths, but in the future, our goal is to establish a direct connection with machine tools for real-time data prediction so that on-site personnel can make adjustments based on future five-axis displacement changes. The training time of the models in this research was time-consuming, and we hope to address this issue in future studies. Long-term time-series problems can lead to model drift, where the performance of the previously trained model deteriorates over time. Therefore, we also aim to develop a system that can effectively address this issue. In addition, this research can be applied to other machine tools.

## Figures and Tables

**Table 1:** Dataset Introduction.

Machine Tool	Operating Conditions
Tool 1	Spray water to heat 10 degrees
Tool 1	Spindle 2350RPM-turn 8 stop 2
Tool 1	Water spray heating 10 degrees-spindle 2350RPM-turn 8 stop 2
Tool 2	Room temperature plus 15 degrees
Tool 2	Room temperature plus 15 degrees-spindle 2350RPM-turn 8 stop 2
Tool 2	Room temperature plus 15 degrees-water spray heating 10 degrees
Tool 2	Room temperature 20 degrees-spindle 2350RPM machine

## Data Availability

The data presented in this study are available on request from the corresponding author due to privacy concerns.
